# Predictive modelling employing machine learning, convolutional neural networks (CNNs), and smartphone RGB images for non-destructive biomass estimation of pearl millet (*Pennisetum glaucum*)

**DOI:** 10.3389/fpls.2025.1594728

**Published:** 2025-05-06

**Authors:** Faten Dhawi, Abdul Ghafoor, Norah Almousa, Sakinah Ali, Sara Alqanbar

**Affiliations:** ^1^ Agricultural Biotechnology Department, College of Agricultural and Food Sciences, King Faisal University, Al Ahsa, Saudi Arabia; ^2^ Center for Water and Environmental Studies, King Faisal University, Al-Ahsa, Saudi Arabia; ^3^ Fab Lab, Abdulmonem Al Rashed Humanitarian Foundation, Al-Ahsa, Saudi Arabia

**Keywords:** digital agriculture, deep learning, plant monitoring, carbon sequestration, CNN - convolutional neural network

## Abstract

Digital tools and non-destructive monitoring techniques are crucial for real-time evaluations of crop output and health in sustainable agriculture, particularly for precise above-ground biomass (AGB) computation in pearl millet (*Pennisetum glaucum*). This study employed a transfer learning approach using pre-trained convolutional neural networks (CNNs) alongside shallow machine learning algorithms (Support Vector Regression, XGBoost, Random Forest Regression) to estimate AGB. Smartphone-based RGB imaging was used for data collection, and Shapley additive explanations (SHAP) methodology evaluated predictor importance. The SHAP analysis identified Normalized Green-Red Difference Index (NGRDI) and plant height as the most influential features for AGB estimation. XGBoost achieved the highest accuracy (R^2^ = 0.98, RMSE = 0.26) with a comprehensive feature set, while CNN-based models also showed strong predictive ability. Random Forest Regression performed best with the two most important features, whereas Support Vector Regression was the least effective. These findings demonstrate the effectiveness of CNNs and shallow machine learning for non-invasive AGB estimation using cost-effective RGB imagery, supporting automated biomass prediction and real-time plant growth monitoring. This approach can aid small-scale carbon inventories in smallholder agricultural systems, contributing to climate-resilient strategies.

## 1 Introduction

In sustainable agriculture, digital technologies and non-destructive monitoring methods are essential for real-time assessments оf crop health and productivity. Biomass serves as an indicator оf crop vigor, reflecting vital processes such as photosynthesis, energy transfer, and water exchange with the atmosphere (Yue et al., 2017; Liu et al., 2022; Zheng et al., 2023). Accurate biomass estimation іs critical for resource allocation, and yield prediction, all оf which contribute tо food security and agricultural production optimization (Niu et al., 2019; Jimenez-Sierra et al., 2021; Tang et al., 2022; Chiu and Wang, 2024). A biomass assessment offers valuable information about carbon sequestration mechanisms since biomass is directly related to the amount of carbon stored in vegetative matter (Lorenz and Lal, 2014; Ortiz-Ulloa et al., 2021; Funes et al., 2022; Chopra et al., 2023; Saleem et al., 2023). In the present study, we emphasized pearl millet (Pennisetum glaucum) which is an important grain crop known for its high nutritional value, climate resilience, and potential health advantages. Its gluten-free characteristics and high nutrient content have inspired the development of numerous food products, like as beverages and infant foods, which are increasingly popular in health-conscious markets around the globe (Deevi et al., 2024). The crop’s remarkable tolerance to hot and arid conditions makes it especially suited to regions facing climate variability, positioning it as a critical player in climate-resilient agriculture and food security (Taylor, 2015; Shrestha et al., 2023). Furthermore, pearl millet has a high potential for carbon sequestration via biomass and soil interactions (Terefe et al., 2020; Ali et al., 2021).

Conventional techniques of biomass measurement are manual, time-consuming, and destructive (Fry, 1990; Bazzo et al., 2023). In recent times, methods for monitoring without causing damage have gained significant attention in research. Thanks to advancements in computer vision, image-based methods are becoming popular for non-destructive monitoring of crop development. These methods specifically take low-level features from digital images and connect them to growth-related phenotypes like leaf area index and biomass (Fry, 1990; Radloff and Mucina, 2007; Tsaftaris et al., 2016; Bazzo et al., 2023). RGB imaging, which uses red, green, and blue wavelengths to record intricate morphological information, has become a potent tool for plant health monitoring (Kefauver et al., 2015; Alves et al., 2021). RGB imagery’s semantic segmentation allows for accurate vegetation categorization by differentiating plant components from backgrounds like soil or debris (Zhuang et al., 2018). Contemporary developments use deep and shallow machine learning to increase segmentation precision; for example, U-net designs identify vegetation from the background, and Support Vector Machines (SVM) categorize vegetation into green and senescent groups based on colour space analysis (Kolhar and Jagtap, 2021; Karthik et al., 2022; Madec et al., 2023).

The introduction of deep learning technologies has fundamentally changed the approaches used in plant development analysis, especially when it comes to the application of sophisticated methods like RGB image segmentation in conjunction with the evaluation of different vegetation indices (Khaki et al., 2020; Zheng et al., 2022). Convolutional neural networks (CNNs) are a cutting-edge deep learning technique that adeptly process images to learn complex features. When provided with a generous amount of data, CNNs are capable of reaching a level of precision that surpasses traditional methods. Consequently, Convolutional Neural Networks (CNNs), for instance, are particularly effective in agricultural applications to extract and interpret complex features related to plant morphology, texture, the diagnosis of plant diseases, detection and enumeration of plant organs, crop yield, and chromatic properties (Castro et al., 2020; Poley and McDermid, 2020; Khaki et al., 2020; Morbekar et al., 2020; Schreiber et al., 2022; Kaya and Gürsoy, 2023; Gonten et al., 2024; Issaoui et al., 2024). Convolutional Neural Networks (CNNs) permit scalable, non-intrusive biomass evaluation, crucial for ecological surveillance, forestry, and precision farming (LeCun et al., 2015; Nakajima et al., 2023; Gülmez, 2024).

Estimating plant biomass from photographs has been attempted in some published papers (Schreiber et al., 2022; Ma et al., 2023; Zheng et al., 2023). One approach involved using linear modelling, hyperspectral imagery, and other vegetation indexes to predict wheat biomass (Yue et al., 2017). Features related to grass growth were evaluated using digital image analysis., Researchers established correlation between observed values of dry matter content, oven-dried biomass, and aboveground fresh biomass with the image-derived attributes, such as the percentage of green pixels and projected area (PA) (Tackenberg, 2007). Additionally, the visual atmospherically resistant index (VARI) and excess green (ExG), two vegetation indices extracted from digital images, were used to evaluate biophysical characteristics of maize. The results showed that ExG effectively estimated overall LAI, while VARI corresponded with green LAI (Sakamoto et al., 2012). Convolutional neural networks (CNN) effectively monitored greenhouse lettuce growth and predicted metrics such as leaf fresh weight (LFW), leaf dry weight (LDW), and leaf area (LA) (Zhang et al., 2020; Gang et al., 2022). Han et al (Han et al., 2019). focused on estimating maize’s above-ground biomass (AGB). They extracted spectral and structural information from photographs using four machine-learning algorithms: artificial neural networks, random forests, support vector machines, and multiple linear regression. The best-performing model was the random forest model, which had low error rates and high explained variance.(Yang et al., 2023). employed the Photosynthetic Accumulation Model (PAM) in combination with a range of vegetative indices to measure the aboveground biomass (AGB) of rice. The results demonstrated that combining height data and VIs produced more accurate AGB projections, providing a reliable technique for evaluating rice growth variables, with R2 values above 0.8. (Wang et al., 2022b). addressed the challenge of predicting above-ground biomass (AGB) in winter wheat at field-scale, finding that random forest models outperforming partial least squares regression models in both training and validation datasets. (Carlier et al., 2023). investigated the application of convolutional neural networks (CNNs) for forecasting biophysical variables in wheat, identifying EfficientNetB4 and Resnet50 as particularly proficient in predicting biomass and nitrogen traits. Their findings indicated that while pseudo-labelling enhanced CNN efficacy, traditional methods like Partial Least Square regression (PLSr) were comparatively less effective, highlighting the need for further research to optimize CNN applications in crop phenotyping.

While artificial intelligence (AI) has been used in agriculture for tasks like disease classification (Kumari et al., 2022), there is still much to learn regarding the utilization of machine learning algorithms for regression analysis tasks. Most prior studies have focused on biomass estimation in major crops such as wheat, rice, maize, and lettuce (Niu et al., 2019; Zhang et al., 2020; Gang et al., 2022; Carlier et al., 2023; Chiu and Wang, 2024). This study targets pearl millet, an underrepresented and a climate-resilient crop critical for arid and semi-arid regions, where precise biomass estimation is essential for sustainable agriculture. This study aims to demonstrate the benefits of utilising machine learning (e.g., XGBoost) and deep learning (e.g., CNN) for measuring above-ground biomass (AGB) of pearl millet. We emphasize its distinct contributions, including a comprehensive evaluation of several vegetation indices (VIs) for AGB estimation and a comparison between the interpretability of XGBoost and CNN’s feature extraction. CNN’s superiority over conventional techniques in identifying intricate correlations in low-dimensional data serves as justification for its use. Furthermore, unlike traditional remote sensing platforms (e.g., UAVs or satellites) which are indeed suited for broader and large scales, our focus on smartphone imaging holds immense potential for democratizing data collection, particularly in scenarios where resources or infrastructure for advanced imaging technologies are limited. Its utility extends to applications such as precision monitoring in small-scale agricultural plots and localized environmental assessments. This approach would bridge the gap between advanced technologies and resource-limited farming communities as well as between small- and large-scale imaging systems by devising cost-efficient models utilizing visible imaging. Additionally, the significance of the study lies in how the integration of smartphone imaging with machine learning can contribute to sustainable agricultural practices by enabling real-time, on-site biomass estimation, which clearly establish the value and relevance of our research to both the scientific community and its practical applications for pearl millet cultivation and facilitating effective crop health monitoring.

## 2 Materials and methods

### 2.1 Experimental design and data collection

This study was structured using a completely randomized block design in a factorial format to evaluate the effects of different treatments on pearl millet growth. The experimental setup included three distinct treatment groups: control, Fertilizer 1, and Fertilizer 2, each with six replicates, totalling 18 experimental units per group. Pearl millet seeds were soaked at 28–30°C for four days to initiate germination. Following germination, 10 seeds were planted in each PVC pot, with pot dimensions of 150 × 100 × 125 mm. Each pot had four drainage holes at the base, and trays were placed underneath to collect drainage. The irrigation schedule, conducted every three days, was determined based on typical meteorological conditions in Saudi Arabia’s Eastern region. A 5-5-5 (N-P_2_O_5_-K_2_O) liquid fertilizer was applied at a concentration of 5 mL per liter of irrigation water. Throughout the experiment, plant height (from ground level to the shoot tip) and dry weight (obtained after drying at 105°C for 24 hours) were recorded at each growth stage (16, 34, 45, 65, and 90 days after sowing) for 10 randomly selected experimental units, providing heterogenous growth metrics throughout the experiment. As a result, 150 points were obtained from all of the readings and used in the data analysis. The following formula was used to convert AGB per pot to Mgha-1


$$AGB\;\left(Mgha^{-1}\;\right)=\frac{AGB\;per\;pot\;\left(g\right)}{100\times Area\;of\;pot\left(m^{2}\right)}\;$$

To facilitate digital analysis, high-resolution RGB images of the plants were captured at each growth stage using an iPhone 14 Pro Max (Apple Inc.). The iPhone was set up one meter above the vegetation. Initially, the digital photographs had pixel resolutions of 900 x 1600. The iPhone’s back camera is 48 MP and has an aperture of 1.78. Every image was taken in a laboratory environment with 700–720 lux of lighting. Images were saved in JPG format with a resolution of 1000 × 800 pixels, forming the primary dataset for subsequent machine learning-based biomass estimation.

### 2.2 Data preprocessing

Data preprocessing was critical to ensure model robustness and enhance its generalization capability. Several pre-processing procedures are applied to the images to improve their quality and prepare them for deep learning model training. For instance, through normalization, the images’ pixel intensity values were scaled to a standard range, such as 0 to 1 or -1 to 1. The photos were resized to a consistent size of 800x800 pixels to guarantee consistency. Various data augmentation techniques including random horizontal and vertical flips, rotations, and modifications in brightness and contrast were applied as standard procedure to artificially increase the diversity of the dataset [44, 48]. Image processing tasks, such as image reading, cropping, and preparation for model training, were executed using Python 3.10.12, employing OpenCV, PIL, Numpy, and Scikit-Image libraries (Van Der Walt et al., 2014; Harris et al., 2020; Sharma et al., 2020). These preprocessing steps aimed to create a diverse and well-prepared dataset, enhancing the deep learning model’s ability to accurately predict biomass and adapt to variances in image quality and environmental conditions.

### 2.3 Semantic segmentation with deep learning

In image analysis, semantic segmentation classifies each pixel in an image using Convolutional Neural Networks (CNNs), which capture intricate spatial correlations. CNNs function by iteratively applying convolutional kernels, which operate through piece-wise multiplication with input data. By combining neighbouring pixel values into a full spatial representation, CNNs can grasp intricate spatial interactions because of their layered structure. Increasingly complex feature representations are created as data moves through these convolutional layers, leading to network topologies customized for particular uses (Kolhar and Jagtap, 2021; Madec et al., 2023). Fully convolutional segmentation workflows generally follow a two-stage approach. First, the model extracts high-level features from input images, encoding significant spatial and textural details. Subsequently, these features are used to predict each pixel’s class at the original resolution. A prominent model for this is the encoder-decoder architecture. In this architecture the encoder condenses the input into a compact, high-level feature representation, retaining essential spatial information. The decoder then reconstructs these features back to the original resolution, ensuring precise spatial alignment of segmented output with the input (Khaki et al., 2020; Kaya and Gürsoy, 2023).

**Figure 1:**
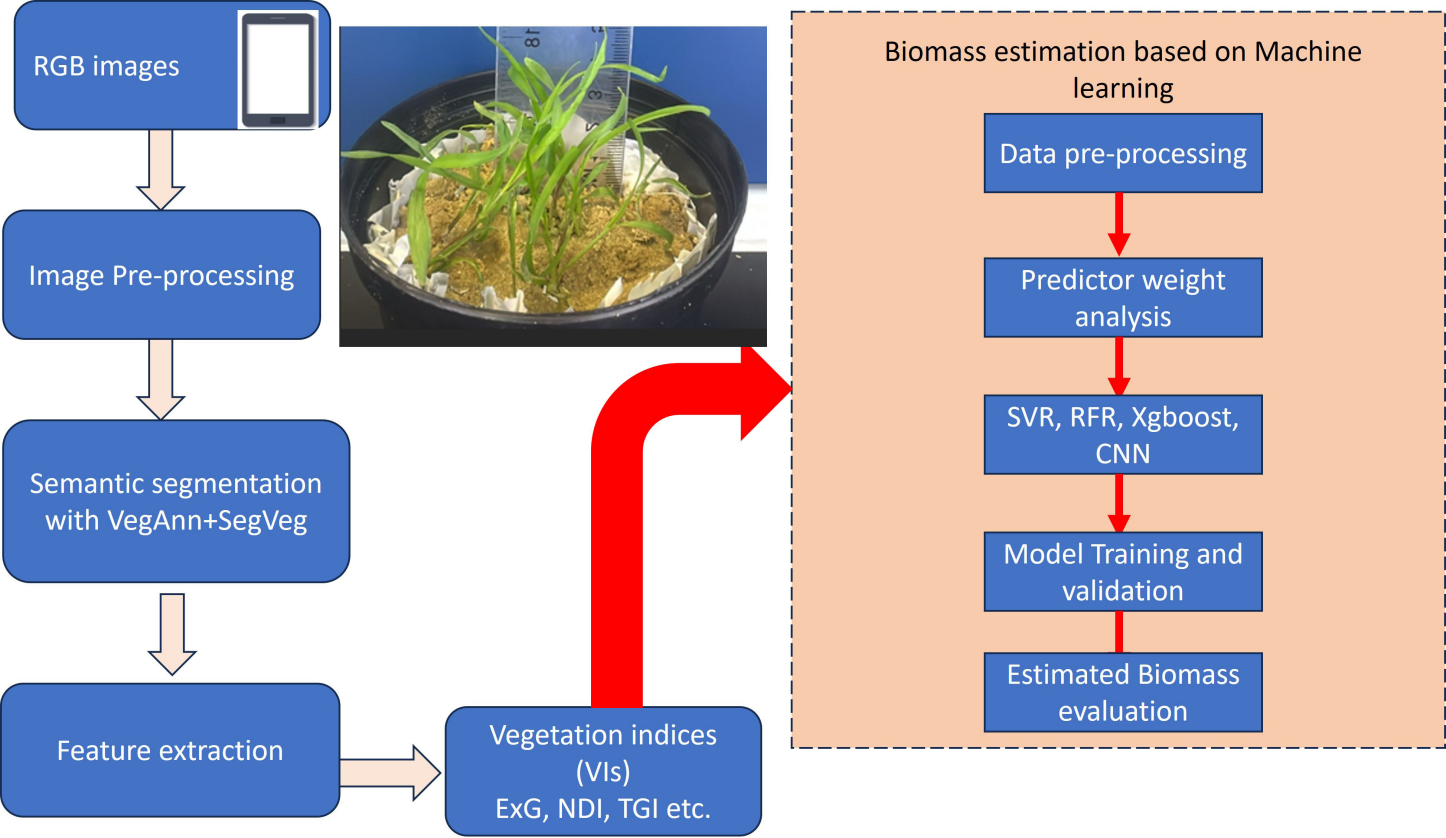
Schematic diagram of the methods employed in this investigation.

In this study, we used weights from the VegAnn dataset to initialize the SegVeg model, an architecture created for plant image segmentation (Serouart et al., 2022), **Figure 1**. Masks were generated using the SegVeg model, which operates in two stages: the first stage uses a U-net architecture to predict binary masks separating vegetation from the background, while the second stage applies a Support Vector Machine (SVM) to classify vegetation pixels into green and senescent categories. This classification capability is vital for ecological and agricultural applications, as it facilitates comprehensive assessments of vegetation cover, plant vitality, and various agronomic factors. The SegVeg model builds on the U-net architecture, a robust encoder-decoder framework tailored for semantic segmentation tasks. U-Net’s structure captures both fine details and broader contextual information, making effective segmentation of complex vegetation patterns. A Support Vector Machine (SVM) layer is integrated, improving the segmentation of plant pixels into green and senescent categories (Serouart et al., 2022; Madec et al., 2023).

The proposed U-Net architecture was implemented, utilizing PyTorch version 2.4 (Facebook, Inc., CA, USA), within the dynamic environment of Google Colab (Google Colab website (last accessed 22 October 2024): https://colab.research.google.com/). An NVIDIA Tesla T4 GPU with 16 GB of dedicated memory was used for its high-performance computing capabilities. The two primary routes of the U-Net architecture are the encoder (down-sampling) and the decoder (up-sampling), which are joined by a bottleneck. There are two convolutional layers and a max-pooling layer for down-sampling in each of the four stages of the encoder pipeline (960 kernels). A 2x2 max-pooling layer cuts the feature maps’ spatial size in half after each step. The encoder and decoder paths are connected by the bottleneck (1024 kernels). Two convolutional layers are used to further process the features that have been extracted. Four up-sampling stages make up the decoder path (960 kernels). To improve spatial resolution, each stage starts with an up-convolution (transposed convolution), which is followed by two convolutional layers. A 2×2 up-convolution (transposed convolution) is used in each up-sampling step to double the spatial dimensions. The final layer uses a 1×1 convolutional layer with 3 kernels to map the output feature maps to the required number of classes (background, green vegetation, and senescent vegetation).

To ensure that each picture could be partitioned into manageable pieces complying to the essential specifications, the original images were selectively padded with zeros as needed, generating consistently dimensioned patches of 800 × 800 pixels that do not overlap. Bias in the training process was eliminated since the plant and background masks were evenly distributed among these patches. All CNN layers had identical feature representations since each mask was normalized to a scale of [0, 1]. To increase the model’s performance and convergence, pre-trained parameters from the ImageNet database were utilized followed by training with the Adam optimizer. Earlier research (Serouart et al., 2022; Madec et al., 2023) provides further detailed descriptions of the model and training procedures. The assessment of semantic segmentation employed a variety of techniques, prominently featuring the intersection-over-union (IoU), accuracy, and F1-score.


$$IOU=\left(\frac{TP}{TP+FP+FN}\right)\times 100$$

$$Accuracy=\left(\frac{TP+TN}{TP+TN+FP+FN}\right)\times 100$$

$$F1-score=100\times \left(\frac{\left(2.TP\right)}{2.TP+FN+FP}\right)$$

**Figure 2:**
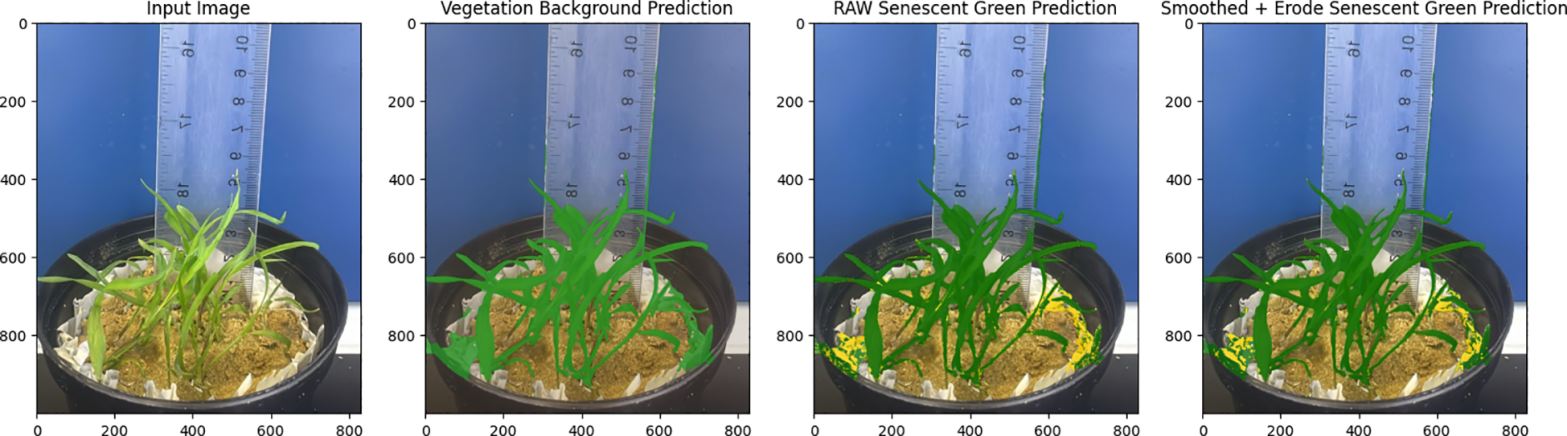
Visualization of image segmentation results of Pearl Millet using VegAnn and SegVeg.

Where TP (true positives) is the proportion of pixels well predicted in green crop class; TN (true negative) is the proportion of pixels well predicted in the background class; FP (false positives) is the proportion of pixels wrongly predicted in green crop class; and FN (false negatives) is the proportion of pixels wrongly predicted in background (**Figure 2**).

### 2.4 Phenotypic feature extraction

RGB images inherently carry a rich spectrum of visual information that may be efficiently used for phenotypic feature extraction. This procedure is critical for extracting and characterizing different visual qualities from picture data. Each RGB picture consists of three spectral channels: red, green, and blue, each represented by a matrix of pixel values that capture different elements of plant shape and health. Based on prior studies demonstrating their significance and utility in estimating AGB (Hamuda et al., 2016; Gang et al., 2022; Gerardo and de Lima, 2023), these indices effectively capture key spectral characteristics of vegetation, such as biomass and chlorophyll concentration, which are intricately associated with AGB. Additionally, indices that minimize sensitivity to external factors, such as soil background and atmospheric interference, were prioritized to enhance model robustness and accuracy. Approximately 16 vegetation indices important to plant growth evaluation were examined (**Table 1**). In this investigation, RGB-based vegetation indices were carefully examined for their ability to evaluate crop biomass. These indices were thoroughly studied as model inputs, allowing for a comparison to determine which indices were the most advantageous. The study’s purpose is to increase the model’s accuracy in predicting phenotypic traits by establishing the optimal indices, providing a reliable framework for analysing plant health and growth dynamics using image-based analysis.

**Table 1:** Definitions of the vegetation index (Vis) extracted from the RGB images of pearl millet.

Vegetation Index*	Equation	Refernces
CIVE (Color Index of Vegetation Extraction)	CIVE = 0.441 × red − 0.811 × green + 0.385 × blue + 18.787	(Kataoka et al., 2003)
Excess Blue Index (ExB)	ExB = 2 × blue−red−green	(Meyer and Neto, 2008)
Excess Green Index (ExG)	ExG=2×green−red−blue	(Woebbecke et al., 1995)
Excess Green minus Excess Red	ExGR=ExG−ExR	(Meyer and Neto, 2008)
Excess Red Index (ExR)	ExR=1.4×red−green	(Meyer and Neto, 2008)
Green Leaf Index (GLI)	$GLI\;=\;\frac{\left(2\times green-red-blue\right)}{\left(2\times green+red+blue\right)}$	(Louhaichi et al., 2001)
Modified Green-Red Vegetation Index (MGRVI)	$MGRVI\;=\;\frac{\left(green^{2}-red^{2}\right)}{\left(green^{2}+red^{2}\right)}$	(Lu et al., 2019)
Normalized Green-Red Difference Index (NGRDI)	$NGRDI=\frac{\left(geen-red\right)}{\left(green+red\right)}$	(Tucker, 1979)
Normalized Difference Index (NDI)	$NDI\;=\;\frac{\left(green-blue\right)}{\left(green+blue\right)}$	(Woebbecke et al., 1995)
RGBVI (Red-Green-Blue Vegetation Index)	$RGBVI\;=\;\frac{\left(green^{2}-red\times blue\right)}{\left(green^{2}+red\times blue\right)}$	(Lu et al., 2019)
Red-Green Ratio Index (RGRI)	$RGRI=\frac{r}{g}$	(Kawashima and Nakatani, 1998)
TGI (Triangular Greenness Index)	TGI = green − 0.39 × red − 0.61 × blue	(Hunt and Daughtry, 2018)
Transformed Green-Red Vegetation Index (TGRVI)	$TGRVI\;=\;\frac{\left(green-red\right)}{\left(green+red\right)}$	(Bannari et al., 1995)
Visible Atmospherically Resistant Index (VARI)	$VARI=\frac{\left(geen-red\right)}{\left(green+red-blue\right)}$	(Gitelson et al., 2002)
Vegetative Index (VI)	VI = green − red	(Tucker, 1979)
Visible Light Vegetation Index (VLVI)	$VLVI\;=\;\frac{\left\{\left(green-blue\right)\right\}}{\left\{\left(green+blue\right)\right\}}-\frac{\left\{\left(green-red\right)\right\}}{\left\{\left(green+red\right)\right\}}$	(Gitelson et al., 2002)

*Vegetation indices are dimensionless ratios of reflectance values measured in specific spectral bands, values typically ranging between -1 and 1 or 0 and 1.

### 2.5 Machine learning regression algorithms

Machine learning regression techniques can effectively describe both linear and nonlinear relationships between predictor variables and crop growth parameters. These methods are especially useful for regression predictions with numerous input variables (Liyew and Melese, 2021; Fragassa et al., 2023; Purushotam et al., 2023; Sharma et al., 2023). We established models to predict the aboveground biomass (AGB) of pearl millet using three shallow machine learning algorithms: Random Forest Regression (RFR), XGBoost, and Support Vector Regression (SVR), as well as one deep learning technique, Convolutional Neural Networks. To assess model accuracy and optimize hyperparameters, we employed a statistical technique called five-fold cross-validation. The process involves randomly dividing a dataset into five subsets, each of which alternates as the test set and the others as the training set. This process is repeated five times to guarantee that all subsets are used completely, and the evaluation metrics from the five tests are then averaged to determine the model’s effectiveness.

A grid search algorithm was utilized to ascertain the optimal hyperparameters. This exploration encompassed a spectrum of parameter configurations, employing five-fold cross-validation and RMSE (Root Mean Square Error) as the principal performance metric. Out of 150 total data specimens, 80% (120 specimens) were designated for training and validation, while the remaining 20% (30 specimens) were reserved as a testing set for evaluating the model’s predictive accuracy on AGB. Python 3.10.12 was used for model creation, feature selection, and data analysis. For regression tasks, the Scikit-learn module (version 1.4) was used, which includes CNN and shallow learning algorithms.

### 2.6 Statistical analysis

The efficacy of each machine learning model was evaluated using a suite of statistical metrics:

Root Mean Square Error (RMSE): Measures the model’s prediction error magnitude, indicating accuracy in predicting AGB.

Coefficient of Determination (R²): Indicates the proportion of variance explained by the model, providing a measure of fit quality. These measurements were crucial for evaluating model performance, determining which model was best for estimating AGB, and confirming each machine learning algorithm’s capacity to improve the accuracy of biomass prediction for pearl millet.


$$RMSE=\sqrt{\frac{1}{n}\sum_{i=1}^{n}{\left(Y_{m}-Y_{p}\right)}^{2}}$$

$$R^{2}=\frac{\sum_{i=1}^{n}{\left(Y_{p}-Y_{av}\right)}^{2}}{\sum_{i=1}^{n}{\left(Y_{m}-Y_{av}\right)}^{2}}$$

where Ym, Yp, and Yav represent the measured value, predicted value, and average value, respectively, and n is the total number of data points.

## 3 Results and discussion

### 3.1 Evaluation metrics of transfer learning approach

Using transfer learning as a component of our methodology, we used a pre-trained model for predictions in this investigation. We specifically employed SegVeg, which was pre-trained on the VegAnn and ImageNet data sets. The basic feature representations were obtained using the pre-training process employed by the model’s original developers. Because the pre-training dataset includes a variety of classes and item categories, the model can pick up strong low-level and mid-level characteristics that apply to our vegetation segmentation problem, like edges, textures, and patterns. Even though our sample data and the pre-training data did not originate from the same source, the features that were acquired from the extensive pre-training dataset are very applicable to the segmentation of vegetation. This ability to generalize has been shown in several picture segmentation tasks, especially for related domains (Trivedi and Gupta, 2021; Karthik et al., 2022; Serouart et al., 2022; Zenkl et al., 2022; Madec et al., 2023). No new pre-training accuracy was produced in this study because our work focusses on employing pre-trained models without further training on the existing dataset. This investigation now demonstrates how well the pre-trained model segments vegetative sections with a high degree of precision. Our test dataset was used to compute quantitative indicators including Mean IoU, F1 score, and pixel accuracy (**Table 2**). A comparison that shows how the pre-trained model performed satisfactorily in segmentation without the need for further fine-tuning.

**Table 2:** Performance metrics for the semantic segmentation.

Metric	Overall Performance	Vegetation	Background
Accuracy	95	94	98
IOU	87	93	90
F1	92	89	91

The model demonstrated strong overall performance in segmenting vegetation and background, achieving 95% overall accuracy, with 94% for vegetation and 98% for background. This suggests that the algorithm properly classifies most pixels, with the background marginally simpler to recognize. The high IoU values indicate a good overlap between predicted and ground truth regions, especially for vegetation, though there is room for improvement in precise boundary delineation. The F1-score shows the model balances precision and recall well but struggles slightly more with vegetation compared to the background. While background is classified with higher accuracy and F1 scores, likely due to its uniform nature and larger representation in the dataset, vegetation performs slightly lower, reflecting challenges in complex textures or overlapping boundaries. These results highlight the model’s reliability but suggest that targeted improvements could further enhance vegetation segmentation performance.

### 3.2 Summary statistics and correlation analysis between vegetation indices

**Figure 3:**
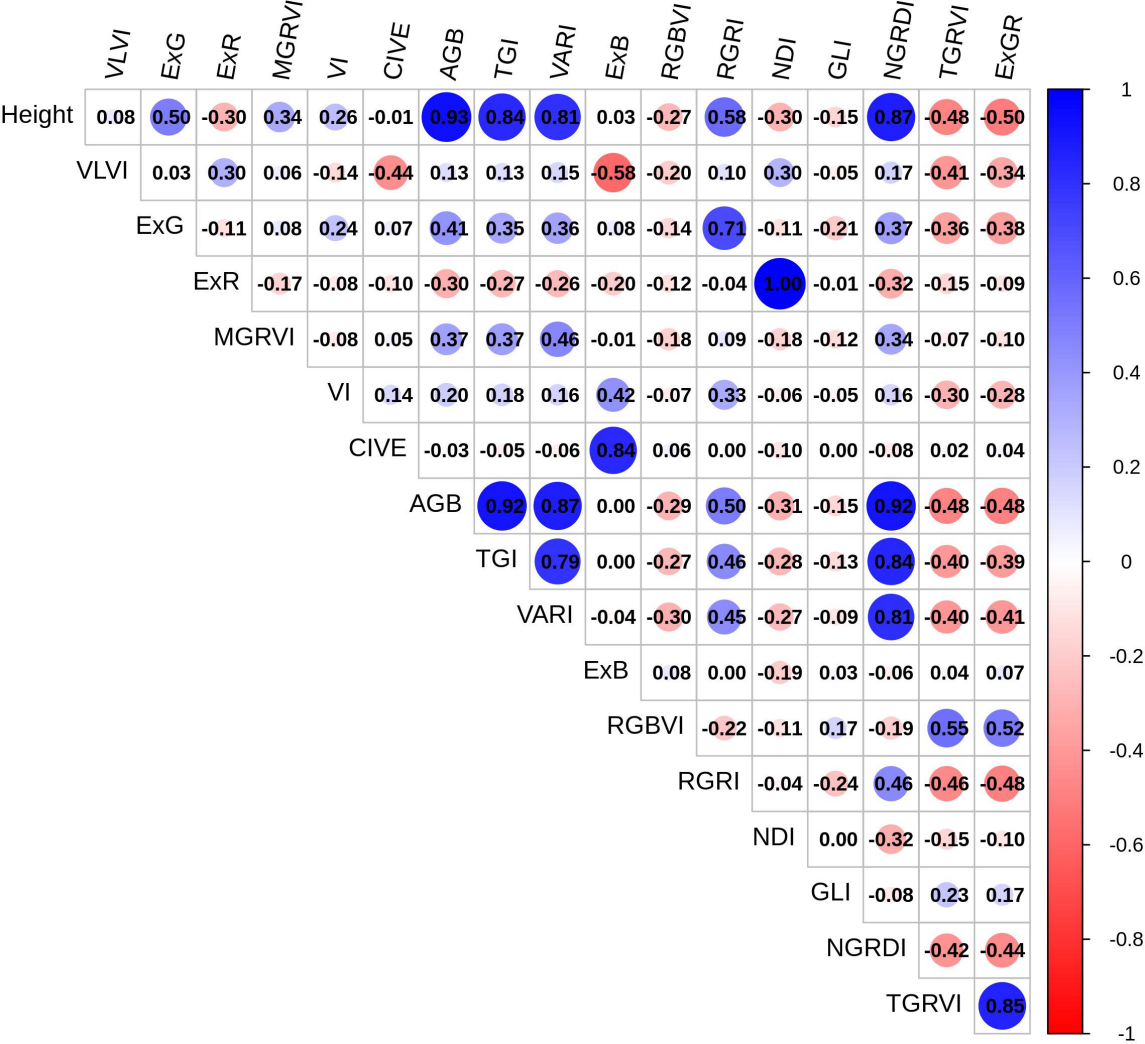
The visualization illustrates a correlation matrix between vegetation metrics and AGB. Red indicates positive correlation, blue indicates negative correlation, and color intensity indicates correlation strength.

**Table 3** summarizes descriptive statistics for various vegetation indices (VI) used to quantify vegetation characteristics. A correlation matrix showing the correlations between several vegetation indices produced from RGB imagery is shown in **Figure 3**. This matrix offers a detailed perspective of the relationships between indices. The largest positive association (0.93) was found between AGB and plant height, followed by NGRDI and TGI (0.92), and VARI (0.87). This suggests that these indices are complimentary for vegetation study since they are sensitive to red and green spectral components. AGB and RGRI and ExG have a somewhat positive relationship, suggesting that their measurements may overlap, perhaps in terms of vegetation structure and greenness. AGB has some sensitivity to indices like MGRVI (0.37) that focus on variations in green reflectance but with less precision than other indices. The weak negative correlation of AGB to RGBVI (-0.29) indicates RGBVI may capture distinct traits, possibly related to background noise (e.g., soil or non-vegetative elements) rather than vegetation structure. There is no correlation between ExB and AGB, suggesting that ExB’s sensitivity to vegetative features does not overlap with that of AGB.

**Table 3:** Summary statistics for the vegetation indices.

Index	Mean	Min	Max	Range	Std
CIVE	0.022	0.154	0.227	0.072	0.021
ExB	0.447	0.079	0.631	0.552	0.159
ExG	0.004	-0.015	0.021	0.036	0.007
ExGR	0.013	-0.006	0.040	0.046	0.011
ExR	0.238	0.001	0.631	0.630	0.159
GLI	0.003	-0.013	0.019	0.032	0.006
MGRVI	0.002	-0.012	0.014	0.026	0.006
NDI	0.118	-0.009	0.318	0.327	0.079
NGRDI	0.011	-0.014	0.037	0.051	0.012
RGBVI	0.004	-0.010	0.021	0.032	0.007
RGRI	0.007	-0.018	0.033	0.052	0.009
TGI	0.011	-0.008	0.039	0.047	0.012
TGRVI	0.021	-0.006	0.059	0.065	0.018
VARI	0.005	-0.006	0.018	0.024	0.006
VI	0.128	0.015	0.184	0.169	0.041
VLVI	-0.001	-0.002	0.000	0.002	0.001

Additionally, ABG and TGI are closely connected, exhibiting similar sensitivity to canopy health and chlorophyll. AGB’s high correlation with height (0.93) and other greenness-related indices (NGRDI, TGI, VARI) makes it a robust choice for monitoring biomass, vegetation health, and canopy coverage. Divergent indices, such as RGBVI, ExB, and ExR, on the other hand, do not match AGB and would be better suited for applications that concentrate on pigmentation or stress. According to these results, a mix of indicators with both high and low correlations may provide a more thorough understanding of vegetation health, structure, and stress response.

### 3.3 Selection of predictors for the AGB biomass estimation using SHAP methodology

In developing predictive models for above-ground biomass (AGB) estimation, the selection of the most relevant predictors is essential to maximize accuracy, reduce noise, and optimize computational efficiency. The Shapley additive explanations (SHAP) technique (Lundberg and Lee, 2017) was used in this investigation to systematically assess predictor significance. A robust interpretive method for machine learning, SHAP assigns significance scores that represent each predictor’s impact on the target variable, allowing for a thorough evaluation of each predictor’s contribution to model predictions (Lundberg and Lee, 2017; Wang et al., 2022a). The SHAP methodology enhances interpretability by highlighting how each feature influences predictions, thereby promoting informed predictor selection. SHAP’s application in this study represents an advancement in the selection process by balancing model interpretability with predictive accuracy. By identifying predictors with the most significant contributions, SHAP aids in isolating those features that most effectively drive AGB predictions, while minimizing the inclusion of uncorrelated or redundant variables. SHAP analysis was performed using the Python package shap. The base model chosen for SHAP analysis in this study was GradientBoostingRegressor from the Python package scikit-learn. All variables were normalized before the SHAP analysis was performed. This was an essential preprocessing step that standardized the scales of the variables to reduce the disproportionate impact of variables with larger magnitudes on the SHAP value calculations.

### 3.4 SHAP analysis results

**Figure 4:**
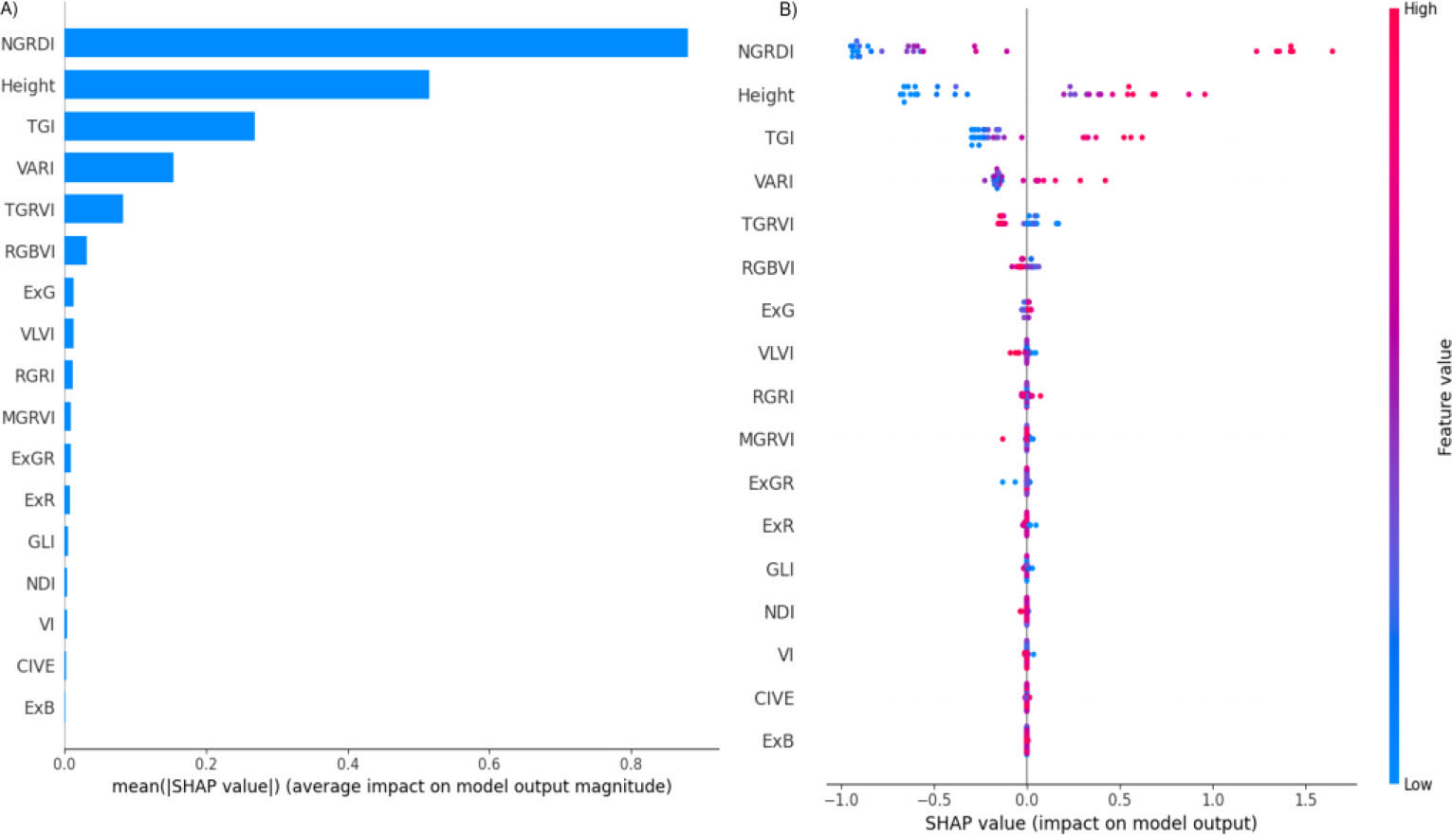
SHAP summary plots display the mean absolute SHAP values for each input feature, representing their average contribution to the model’s prediction of above-ground biomass (AGB). Panel **(A)** ranks the characteristics based on their overall influence, with higher values indicating greater impact on AGB projections. Panel **(B)** offers a more granular view, illustrating how individual feature values (represented by colored dots) influence the model output in both positive and negative directions. The color gradient in Panel **(B)** reflects the magnitude of the feature value (from low to high), enabling interpretation of whether high or low values of a characteristic tend to increase or decrease AGB predictions across all instances.

**Figure 4** provides two visual SHAP representations, offering insights into the significance and impact of each predictor on model outputs. **Figure 4A** illustrates the average SHAP values assigned to each feature, representing the mean effect of each feature on AGB predictions.

-NGRDI (Normalized Green-Red Difference Index): NGRDI exhibits the highest average SHAP value, indicating it as the most influential predictor. As a commonly used index in vegetation analysis, NGRDI likely has a strong correlation with AGB, underscoring its critical role in the model’s predictive framework.

Height: Height is ranked as the second most important predictor and has a strong correlation with AGB. This suggests that plant height is directly associated with biomass accumulation, and therefore contributes substantially to model predictions.

TGI (Triangular Greenness Index) and VARI (Visible Atmospherically Resistant Index): These indices follow NGRDI and height in importance. Although their impact is relatively smaller, TGI and VARI contribute valuable information on vegetation greenness, which is pertinent to biomass estimation.

TGRVI (Triangular Green-Red Vegetation Index) and RGBVI (Red-Green-Blue Vegetation Index): These indices demonstrate a moderate influence on model predictions but are less critical than the top-ranked features. They still provide additional insights that may enhance the model’s accuracy by offering different perspectives on plant vigor and color differentiation.

Less Significant Predictors (e.g., ExG, VLVI, RGRI): These features exhibit minimal average SHAP values, indicating a low contribution to AGB predictions. Their limited impact suggests that they may be less relevant or potentially redundant for this specific model, offering little additional predictive power.

The SHAP analysis confirms that the most influential features (such as NGRDI and height) substantially contribute to AGB estimation accuracy, whereas features with low SHAP values may be excluded from the final model to streamline computations without compromising predictive performance. This selective approach, informed by SHAP, enhances model efficiency by concentrating on high-impact predictors, which ultimately improves both the interpretability and accuracy of AGB predictions.

**Figure 4B** (also called Bee Swarm Plot) offers a more in-depth perspective on how the value of each feature affects the output of the model. Each point depicted in this visualization corresponds to a SHAP value for a specific feature within a single observation, and the points are color-coded based on the feature’s value, ranging from blue for low values to red for high values. The analysis indicates that high values (represented in red) tend to positively influence the model’s output, effectively pushing it to the right, while low values (shown in blue) appear to have a detrimental effect. This observation suggests that elevated NGRDI values are likely associated with higher predictions, which may indicate the presence or overall health of vegetation in the assessed area. Similar to the NGRDI trend, high values of height (represented by red points) generally result in higher model output, implying that taller plants might correlate with higher predictions, thus highlighting the importance of height in the predictive model. The influence of TGI on the model is somewhat more complex and nuanced. While elevated values (red) generally exert a positive impact, the consistency of this effect appears to be less reliable when compared to the clear trends associated with NGRDI or Height. VARI feature exhibits a varied spread of impact values, indicating that both high and low values of VARI can significantly affect the model’s output, although the overall influence seems to be comparatively smaller in magnitude. For those features that register very low SHAP values (for instance, ExG, VLVI, MGRVI), the points are densely clustered around zero. The fact that the points for many of the features that are considered less significant are closely clustered around zero emphasizes that they contribute very little to the model’s output and are typically unaffected by changes in high or low values.

### 3.5 Estimation of AGB based on machine learning algorithms

**Figure 5:**
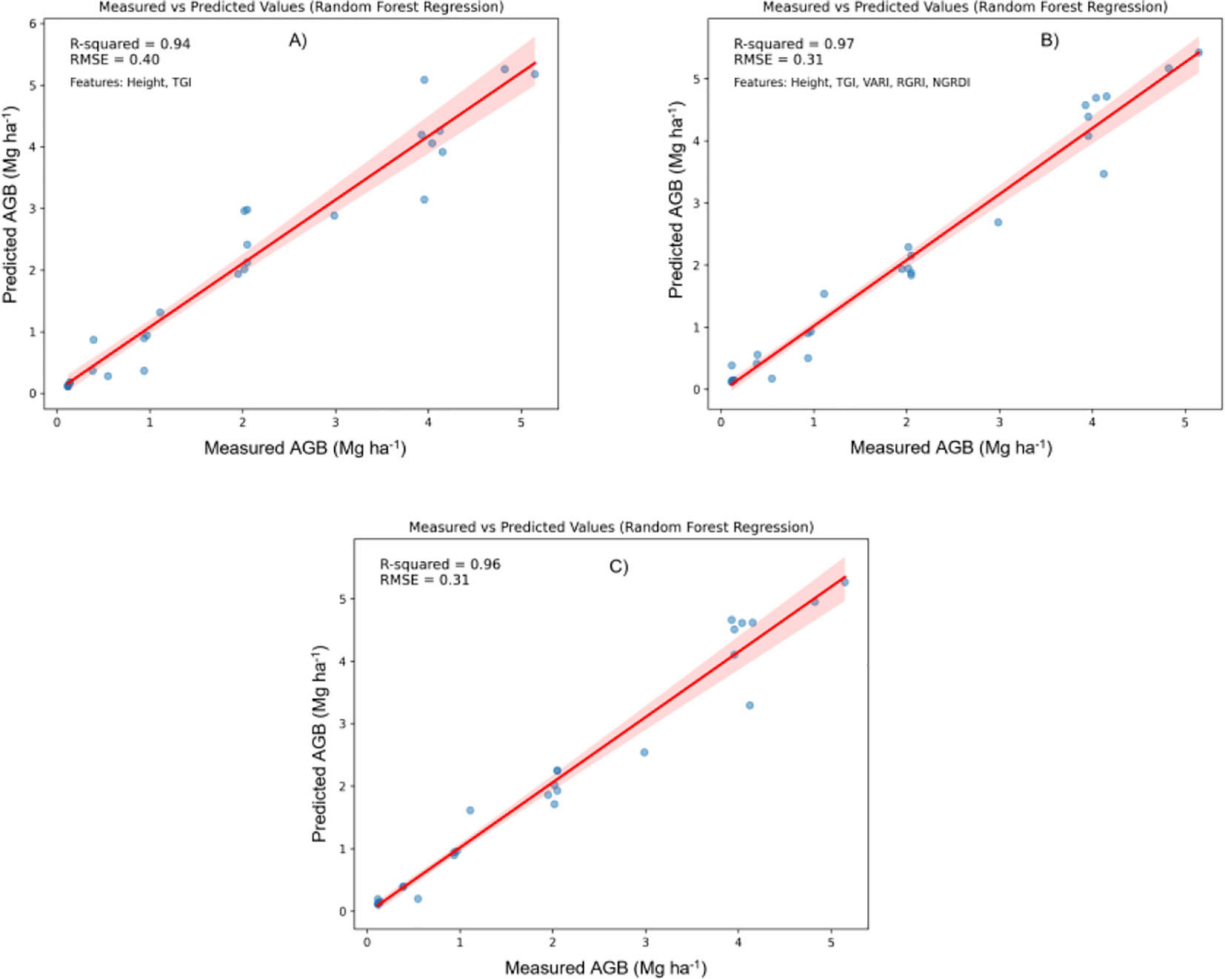
Relationship between measured AGB and predicted AGB using random forest regression (RFR). **(A)** Two best features; **(B)** Five best features; **(C)** all features.

**Figure 6:**
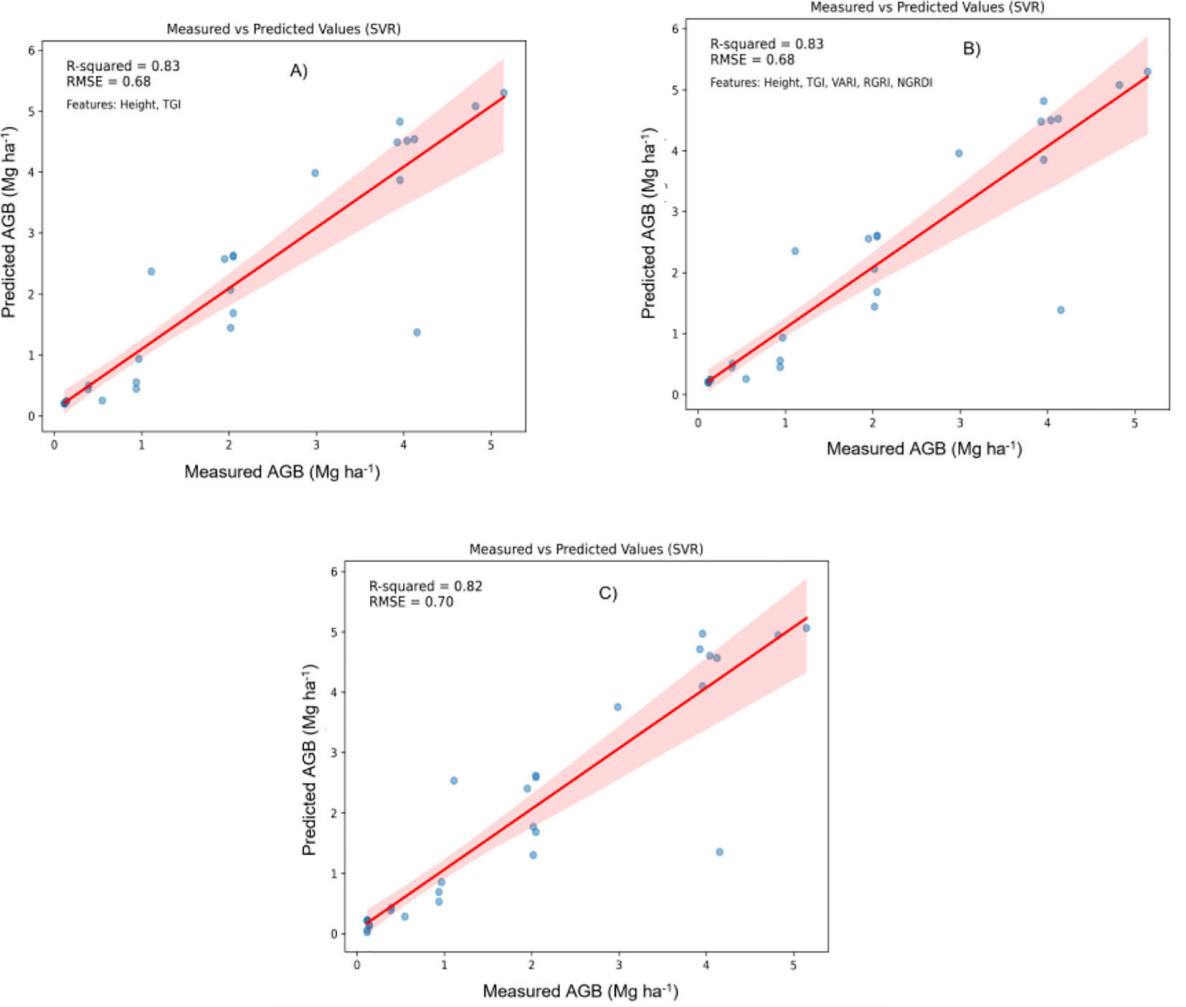
Relationship between measured AGB and predicted AGB using the Support Vector regression (SVR) model. **(A)** Two best features; **(B)** Five best features; **(C)** all features.

**Figure 7:**
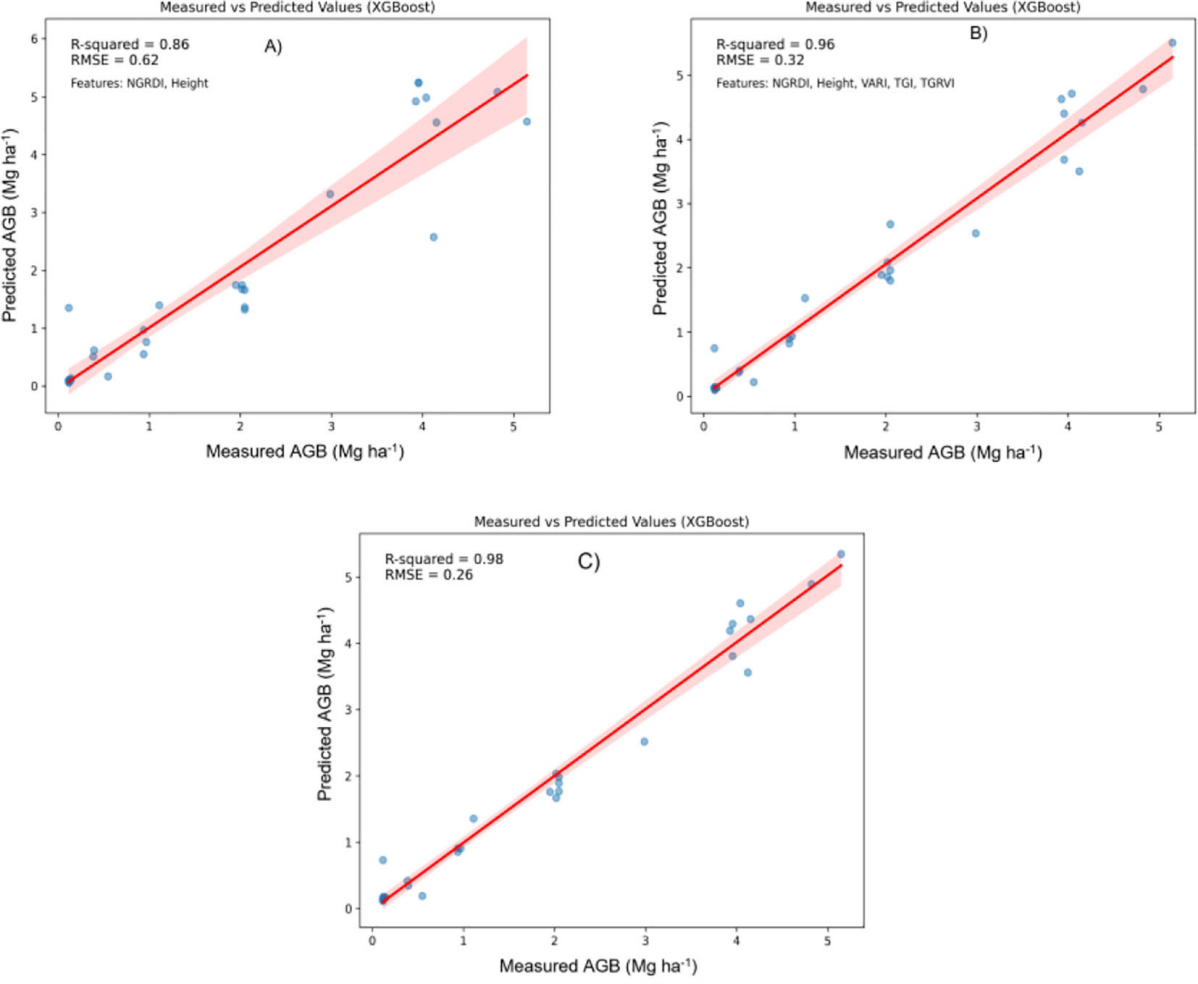
Relationship between measured AGB and predicted AGB using the Extreme Gradient Boosting (XGBoost) model. **(A)** Two best features; **(B)** Five best features; **(C)** all features.

In this study, Machine Learning (ML) techniques were applied to estimate the above-ground biomass (AGB) of Pearl millet, utilizing both shallow and deep learning models. ML methodologies facilitate efficient and precise model development, enhancing predictive accuracy across diverse analytical frameworks. Both shallow algorithms, such as Random Forest Regression (RFR), Support Vector Regression (SVR), and XGBoost, as well as deep learning architectures like Convolutional Neural Networks (CNNs) were examined. Shallow algorithms, characterized by their computational efficiency and enhanced interpretability, provide significant benefits in contexts where resources are constrained. Conversely, CNNs are capable of extracting complex patterns from data, making them powerful but resource-intensive (Zhang et al., 2020; Kolhar and Jagtap, 2021). Metrics such as the Root Mean Square Error (RMSE) and coefficient of determination (R2) were employed to compare machine learning algorithms. **Figures 5**–**7** illustrate scatter plots depicting the predictions of each model, comparing both comprehensive and selected feature sets. These scatter plots show the correlation between predicted and observed AGB values. The results indicate strong predictive performance across models, with R² values ranging from 0.82 to 0.98 and RMSE values from 0.20 to 0.70 Mg ha^1^, reflecting a strong alignment between predicted and actual AGB values.

Support Vector Regression (SVR): As shown in **Figure 6**, SVR demonstrated a broader scatter of sample points, indicating lower precision in AGB predictions. The widespread around the 1:1 regression line suggests that SVR may not capture the underlying relationships as effectively, leading to a relatively less accurate estimation.

Random Forest Regression (RFR) and XGBoost: In contrast, **Figures 5** and **7** show that both RFR and XGBoost predictions are closely grouped around the 1:1 line, highlighting their superior performance in regression tasks. These models exhibit less deviation, indicating greater consistency and precision in predicting AGB. Notably, XGBoost demonstrated the highest accuracy, with R² reaching 0.98 and an RMSE of 0.26 Mg ha^1^, indicating minimal prediction error. The clustering of points around the regression line emphasizes XGBoost’s robust predictive power.

**Figure 7** provides additional insights by comparing XGBoost model predictions using the full feature set and a selective subset of features (NGRDI, Height, VARI, TGI, and TGRVI):

Full Feature Set (**Figure 7C**): The XGBoost model, when utilizing all available features, achieved the highest performance with an R² of 0.98 and an RMSE of 0.26 Mg ha^1^. This configuration closely aligns with the observed values, as indicated by the tight clustering along the red line in the scatter plot.

Reduced Feature Set (**Figures 7A, B**): In these plots, the XGBoost model’s performance with a reduced subset of five selected features is also presented. This configuration yielded an R² of 0.96 and an RMSE of 0.32 Mg ha^1^, a slight reduction in accuracy compared to the full feature model. Nevertheless, this reduced model remains highly effective, maintaining strong alignment with actual AGB measurements. The minimal drop in accuracy suggests that a reduced set of well-chosen features can still provide reliable predictions, particularly in cases where simplicity is prioritized or data constraints exist.

The comparative analysis underscores that incorporating a broader set of features generally improves model accuracy. However, the XGBoost model, using a carefully selected subset of five features, still achieved commendable predictive accuracy. This finding highlights the potential for efficient AGB estimation with fewer predictors, offering a practical solution for scenarios where data availability or computational resources are limited. XGBoost emerged as the most effective model for AGB prediction, demonstrating a strong correlation between predicted and observed values, with minimal error. While incorporating all features maximizes accuracy, the model using the selected five features continues to provide reliable predictions, making it a viable alternative in simplified modelling applications.

### 3.6 Deep learning approach for biomass estimation using convolutional neural networks

CNNs are largely recognized for processing image data, however, recent research has shown that they can also handle low-dimensional and one-dimensional data effectively by using their capacity to extract hierarchical features and patterns. CNNs have been effectively utilized in tasks involving regression and time series analysis (Srivastava et al., 2022; Purushotam et al., 2023; Sharma et al., 2023). This study proposed a deep learning approach using Convolutional Neural Networks (CNNs) to estimate the aboveground biomass (AGB) of Pearl millet. Vegetative variables were processed through a CNN, leveraging 1-dimensional convolution operations to capture complex, nonlinear interactions among input features. Each input variable was processed independently within the same CNN model, and the resulting CNN outputs were concatenated to form a cohesive predictive representation.

The CNN architecture was implemented in Python (version 3.10.12) using the TensorFlow framework (version 2.17.0). Model weights were initialized with a uniform distribution, while biases were initialized to zero, following standard TensorFlow configuration practices. The Adam optimizer was used for training, with Mean Squared Error (MSE) serving as the loss function. The model was trained for a total of 100 and 200 epochs, with each epoch representing a complete pass through the training dataset, during which model weights were iteratively updated. After each epoch, the model’s validation Root Mean Square Error (RMSE) was computed using a reserved validation dataset to monitor performance independently of the training data. Optimal model parameters were identified based on the minimum validation loss achieved during training. Learning rate decay was applied throughout the training process to gradually reduce the learning rate every 20 epochs, helping the model converge to a minimum. The learning rate decay followed a schedule in which the initial learning rate was sequentially multiplied by 0.8, 0.6, 0.4, and 0.2 at each 20-epoch interval, enabling controlled adjustments to the rate of learning. Hyperparameters, including batch size and initial learning rate, were fine-tuned to optimize model performance. Batch size was varied across values of 8, 16, 32, and 64, while the initial learning rate was tested with values of 0.1, 0.01, 0.001, 0.0001, and 0.00001. The optimal combination of batch size and learning rate was determined based on the model’s predictive accuracy on the test dataset. This combination was then used to train the final CNN model, maximizing accuracy for AGB estimation.

**Figure 8:**
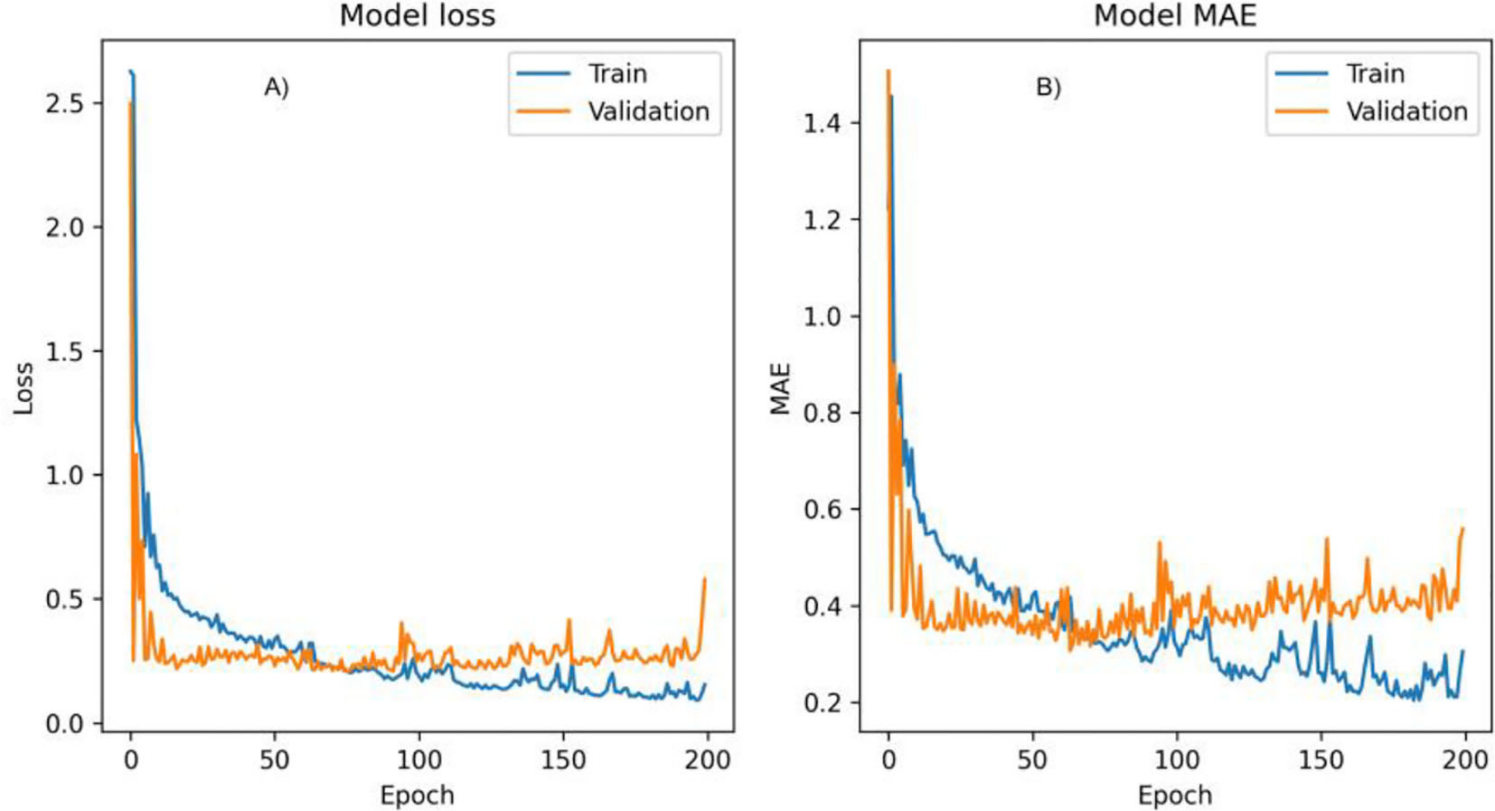
Graph showing the training and validation performance of a model over 200 epochs, showing both loss **(A)** and Mean Absolute Error (MAE) metrics **(B)**.

The most accurate model, based on the optimal batch size and learning rate configuration, was selected to predict Pearl millet biomass. The evolution of training and validation losses over the epochs for this model is illustrated in **Figure 8**. This plot provides insights into the model’s convergence behaviour, showing a consistent reduction in training and validation losses, particularly as the model approaches its optimal configuration. The validation loss trend also demonstrates the model’s capacity to generalize, avoiding overfitting by maintaining stable performance across training iterations.

### 3.7 Training and validation performance analysis

**Figure 8** illustrates the training and validation performance of the model over 200 epochs, providing insights into the model’s behaviour across both loss and Mean Absolute Error (MAE) metrics.

#### 3.7.1 Model loss plot

The loss plot tracks the model’s loss during training (blue line) and validation (orange line) phases. At the beginning of training, both training and validation losses show a sharp decrease, indicating rapid learning as the model adjusts its parameters to minimize prediction error. Following this initial drop, losses stabilize, fluctuating around lower values, with the training loss consistently remaining below the validation loss. This consistent difference suggests that the model fits well with the training data but may be capturing some patterns that don’t generalize perfectly to the validation set. Towards the end of training, a slight increase in validation loss is observed, possibly indicating the onset of overfitting, where the model becomes more tailored to the training data at the expense of generalization.

#### 3.7.2 Model MAE plot

Similar to the loss plot, the MAE plot shows both training (blue line) and validation MAE (orange line). The MAE starts high for both phases but drops quickly in the initial epochs, suggesting that the model is quickly learning to minimize absolute prediction errors. Throughout training, the training MAE remains consistently lower than the validation MAE, which implies that the model is more accurate on the training data than on the validation data. Some variability is present in both training and validation MAE values across epochs, reflecting minor fluctuations as the model fine-tunes its weights. However, towards the end of training, the validation MAE begins to increase slightly, which may also indicate overfitting.

Both the loss and MAE metrics stabilize early in training, suggesting that the model effectively learns the main patterns in the data within the initial epochs. However, the upward trend in validation loss and MAE towards the end implies a slight overfitting tendency as training continues. This could potentially be mitigated by implementing early stopping or by tuning regularization parameters to prevent the model from overfitting. **Figure 8** highlights that while the model rapidly learns and stabilizes, its slight overfitting towards the final epochs suggests a potential area for improvement in future training runs.

**Figure 9:**
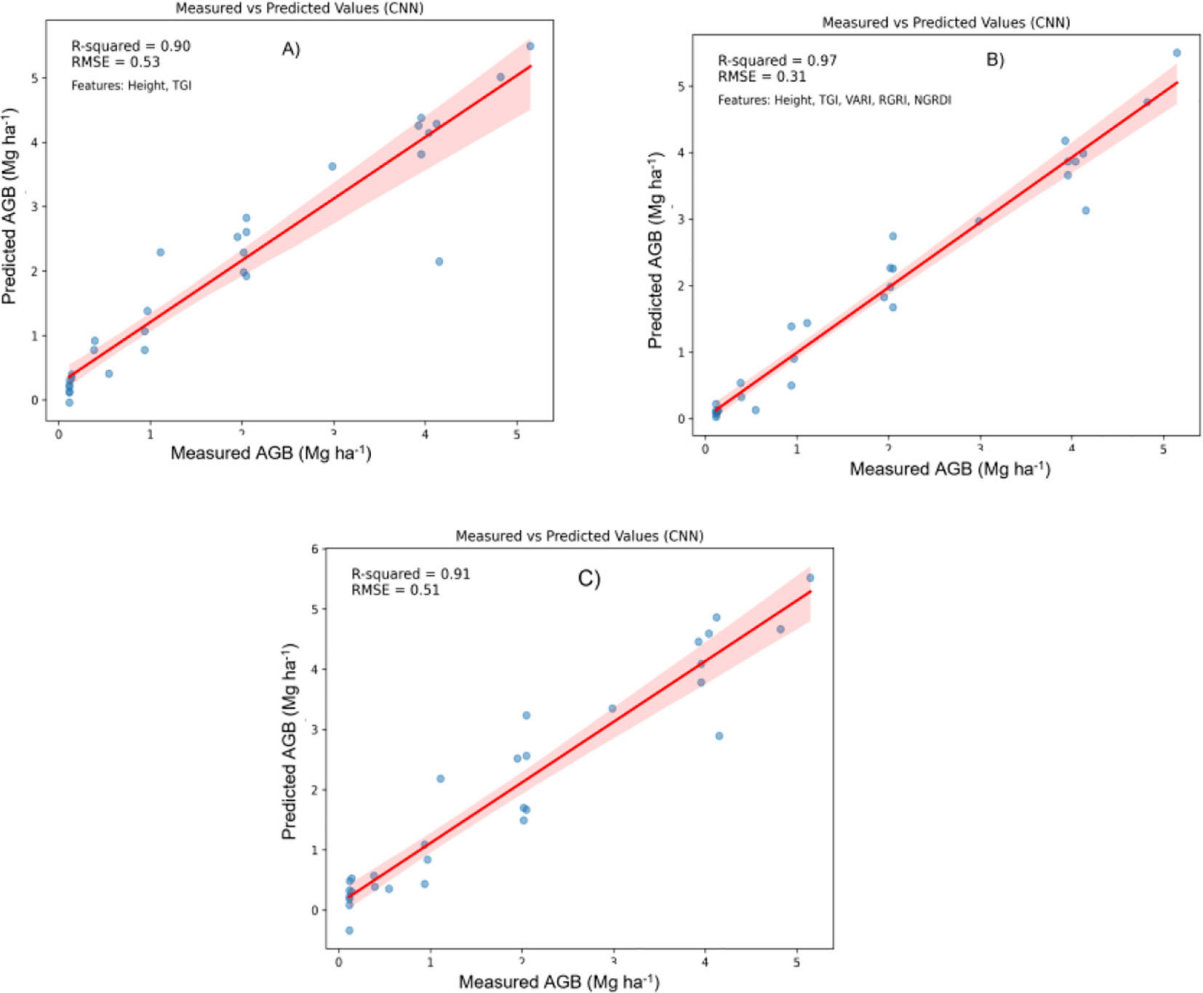
Relationship between measured AGB and predicted AGB using Convolutional neural networks (CNNs) model. **(A)** Two best features; **(B)** Five best features; **(C)** all features.

The predictive accuracy of the CNN model for estimating above-ground biomass (AGB) on the test dataset is presented in **Figure 9**. The model shows high effectiveness, evidenced by substantial correlations between measured and predicted AGB values, showcasing the model’s capacity to accurately predict biomass. In **Figure 9A**, the plot includes height and the Triangular Greenness Index (TGI) as predictors, yielding an R-squared value of 0.90 and a Root Mean Square Error (RMSE) of 0.53. This high R-squared indicates a strong linear relationship between measured and predicted AGB, while the moderate RMSE suggests some error, visualized by the red trendline and associated confidence interval. This configuration demonstrates that height and TGI alone provide a reliable foundation for AGB prediction, though with some degree of residual error.

By incorporating height, TGI, Visible Atmospherically Resistant Index (VARI), Red-Green Ratio Index (RGRI), and Normalized Green-Red Difference Index (NGRDI), this configuration achieves superior predictive performance. The R-squared increases to 0.97, and the RMSE drops to 0.31, indicating a very close match between measured and predicted values. This improvement suggests that these five features capture a broader range of vegetation characteristics, enhancing model accuracy and reducing error. The increased R-squared and lower RMSE indicate that the model effectively leverages additional indices to improve AGB prediction.

In **Figure 9C**, the plot utilizes all available features, resulting in an R-squared of 0.91 and an RMSE of 0.51. While this setup slightly outperforms, **Figure 9A** shows lower performance compared to the configuration in **Figure 9B**. This suggests that adding more features beyond the five used in **Figure 9B** may introduce noise or redundancy, slightly reducing the model’s predictive efficiency.

The integration of five features (height, TGI, VARI, RGRI, and NGRDI) provides the highest predictive accuracy, as shown by the highest R-squared (0.97) and lowest RMSE (0.31) values. This configuration strikes an optimal balance, where the addition of indices beyond height and TGI significantly enhances the model’s predictive power, but including all features yields only a marginal improvement. Each plot demonstrates a positive relationship between measured and predicted AGB, with confidence intervals representing the inherent uncertainty in predictions. Overall, these findings suggest that a focused set of relevant indices can achieve high model performance, with the five-feature configuration proving the most effective for accurate AGB estimation.

### 3.8 Evaluation and comparison of machine learning models for estimating above-ground biomass

**Table 4** provides a comparative analysis of four machine learning models—Random Forest Regression (RFR), Support Vector Regression (SVR), XGBoost, and Convolutional Neural Networks (CNNs)—in estimating AGB using different feature sets: all features, the five most significant features, and the two most salient features. Performance metrics are reported as R² (coefficient of determination) and RMSE (root mean square error).

**Table 4:** AGB estimation accuracy using different machine learning algorithms.

Features	RFR		SVR		XGBoost		CNNs	
	R^2^	RMSE	R^2^	RMSE	R^2^	RMSE	R^2^	RMSE
All features	0.96	0.31	0.82	0.70	0.98	0.26	0.91	0.51
5 best features	0.97	0.31	0.83	0.68	0.96	0.32	0.97	0.31
2 best features	0.94	0.20	0.83	0.68	0.86	0.62	0.90	0.53

All Features: XGBoost achieved the highest accuracy (R² = 0.98, RMSE = 0.26), followed by RFR (R² = 0.96, RMSE = 0.31). CNNs exhibited moderate performance (R² = 0.91, RMSE = 0.51), and SVR was the least effective (R² = 0.82, RMSE = 0.70). These results indicate that XGBoost benefits from the complete feature set, leveraging its robust gradient-boosting framework to minimize errors effectively.

Five Most Significant Features: Both RFR and CNNs demonstrated high accuracy, with R² ≈ 0.97 and RMSE ≈ 0.31. XGBoost performed slightly lower (R² = 0.96, RMSE = 0.32), while SVR continued to lag (R² = 0.83, RMSE = 0.68). The comparable performance of RFR and CNNs with fewer features suggests that they are capable of effective prediction without requiring a full feature set, making them adaptable for resource-constrained scenarios.

Two Most Significant Features: RFR emerged as the top performer, achieving R² = 0.94 and RMSE = 0.20. XGBoost and CNNs showed declines in performance, with CNNs reaching an R² of 0.90 and RMSE of 0.53, while SVR maintained its relatively low performance (R² = 0.83, RMSE = 0.68). This result indicates that RFR retains accuracy even with a reduced feature set, highlighting its flexibility and robustness.

Multivariate features have more predictive power for AGB than single variable features, as shown in earlier investigations (Lu et al., 2019). Similarly, when evaluating biophysical crop parameters, (Han et al., 2019). stressed that vegetation indices of photographs might be taken into account simultaneously rather than separately. Consistent with earlier research, plant height has a major impact on biomass yield, making it a significant agricultural architecture that is strongly connected with biomass yield (Montes et al., 2011; Schirrmann et al., 2016; Naito et al., 2017; Lu et al., 2019; Yu et al., 2023). (Schirrmann et al., 2016). showed that the estimation accuracy could be effectively increased by incorporating plant height into the model designed to estimate AGB. Similar findings were made by (Naito et al., 2017). and (Yu et al., 2023).

### 3.9 Overall summary of model performance

Estimating AGB accurately is essential for understanding carbon sequestration and enhancing agricultural management (Ortiz-Ulloa et al., 2021; Funes et al., 2022; Chopra et al., 2023). XGBoost performs best with a comprehensive feature set, achieving superior predictive accuracy with an R² of 0.98 and RMSE of 0.26. This is attributed to its advanced gradient-boosting approach, which integrates multiple weak learners to minimize errors through sequential corrections (Wang et al., 2022a; Sharma et al., 2023). XGBoost uses a combination of a loss function and a regularization term to prevent overfitting, enhanced by features such as gradient descent optimization, column subsampling, and shrinkage for improved convergence and stability. Additionally, XGBoost’s ability to handle missing data and optimize tree structure through parallel processing adds to its effectiveness. RFR shows superior performance with a reduced feature set, indicating a high level of adaptability. With its ensemble-based structure, RFR combines predictions from multiple decision trees, effectively capturing nonlinear relationships in the data and reducing sensitivity to noise (Chiu and Wang, 2024). This adaptability makes RFR a suitable choice when computational resources are limited or when fewer predictor variables are available. SVR consistently underperforms in comparison to other models across all feature sets (R² = 0.82–0.83, RMSE = 0.68–0.70). As a linear regression model, SVR struggles with the complex nonlinear relationships inherent in the dataset, which limits its accuracy. Its susceptibility to outliers and reliance on linear transformations may explain its lower performance (Liu et al., 2021). CNNs perform moderately well across different feature sets, showing potential for improvement with further tuning (Carlier et al., 2023). CNNs excel at extracting complex features from data, which is beneficial for tasks involving unstructured data (such as images) but may be less suited for purely tabular data where tree-based models like XGBoost and RFR perform better. Overall, the observed differences in performance indicate that nonlinear regression models outperform linear alternatives like SVR, consistent with findings from prior studies (Lu et al., 2019; Nakajima et al., 2023). This is consistent with (Zhai et al., 2023)’s findings that the RFR approach performed better than alternative machine learning algorithms, resulting in increased AGB estimation accuracy.

### 3.10 Limitations and perspectives

Vegetation indices (VIs) are effective for estimating aboveground biomass (AGB) because they are designed to highlight specific spectral features of vegetation that correlate with biomass attributes. Studies have shown that the inclusion of red-edge and NIR bands further improves the sensitivity of indices to variations in biomass and reduces the influence of confounding factors such as soil background and atmospheric conditions (Woebbecke et al., 1995; Hamuda et al., 2016; Yue et al., 2017; Lu et al., 2019)​. The current study focuses on one-dimensional VI combinations, potentially overlooking spatial patterns available in raw spectral imagery. Future studies could explore integrating data from multiple sensors (e.g., hyperspectral and multispectral imagery) which could enhance the accuracy and robustness of AGB estimation. Investigating newer indices tailored for specific vegetation types or environmental conditions could address limitations in biomass estimation under extreme conditions.

XGBoost emerged as the best-performing model, with high R² and low RMSE values. Key features contributing to XGBoost’s success include its capability to aggregate weak learners, implement gradient boosting, and minimize an objective function that combines a loss function with regularization. However, XGBoost’s computational demands are significant, requiring substantial memory and processing power, particularly for large or high-dimensional datasets. The extensive hyperparameter tuning required for XGBoost further necessitates considerable expertise and computational resources. Moreover, XGBoost is less effective for unstructured data, such as images, where deep learning architectures like CNNs generally perform better. Moreover, this study primarily focuses on one-dimensional data regression analysis and does not explore two-dimensional data analysis methods for AGB prediction modelling. This limits the potential advantages of using CNN over XGBoost, as CNNs are specifically well-suited for extracting spatial features from two-dimensional data.

This study’s methodology highlights the potential for farmers to use accessible technologies, such as smartphone devices, and enter them into automated biomass prediction algorithms, which could serve as the foundation for carbon sequestration inventories. The integration of machine learning algorithms into agricultural practices could facilitate the creation of automated AGB estimation systems, providing accurate biomass predictions essential for precision agriculture (Lu et al., 2019; Fragassa et al., 2023; Purushotam et al., 2023; Sharma et al., 2023). Even with noise-prone image data, the models demonstrated satisfactory accuracy, supporting the feasibility of using smartphone-acquired images for AGB estimation in field setting. The current study focuses on a single crop variety under controlled growth conditions, which was a deliberate choice to ensure the feasibility of the study within the given scope and resources. Future research could also explore the application of this framework on a larger scale by incorporating data from diverse environmental conditions, multiple crop varieties, crop growth stages, and geographical regions to assess the model’s generalizability. Expanding the dataset to include multispectral or hyperspectral imagery could further improve model accuracy by capturing additional vegetation characteristics. Integrating real-time monitoring systems through IoT-enabled sensors with machine learning models could provide continuous, automated AGB estimations, aiding decision-making in dynamic agricultural environments. Lastly, implementing transfer learning with other advanced neural networks or refining hyperparameters may enhance the predictive performance of CNNs, particularly in estimating biomass in unstructured and complex agricultural settings.

## 4 Conclusions

In conclusion, this study proposes a non-destructive framework for forecasting above-ground biomass (AGB) in pearl millet by combining Convolutional Neural Networks (CNNs) with shallow machine-learning methods. The results show that sophisticated deep learning approaches like CNNs, when combined with machine learning, particularly tree-based algorithms like XGBoost, may provide reliable AGB predictions. XGBoost outperformed other models when a comprehensive feature set was utilized, achieving the highest R² and the lowest RMSE values. Random Forest Regression (RFR) demonstrated effectiveness with reduced feature sets, highlighting its versatility and efficacy under data-constrained scenarios.

In summary, this study emphasizes the potential of readily available digital technologies, such as smartphone-acquired photos, to support automated biomass prediction and real-time crop monitoring. The unique characteristics of small farming systems, such as diverse cropping patterns, agroforestry practices, and integrated livestock, play a crucial role in carbon dynamics. These tools could form the basis for small-scale carbon inventories to measure the carbon sequestered within vegetation biomass in smallholder agricultural systems. These inventories are crucial for understanding how agricultural practices contribute to carbon sequestration at the local level and for informing climate-resilient strategies.

## Data Availability

The original contributions presented in the study are included in the article/**Supplementary Material**. Further inquiries can be directed to the corresponding authors.
